# Supplementary material for: EnzML: multi-label prediction of enzyme classes using InterPro signatures
Source: BMC Bioinformatics. 2012 Apr 25;13:61. doi: 10.1186/1471-2105-13-61 (PMC3483700; doi:10.1186/1471-2105-13-61)
Supplement: Addtional file 5 — The Java code to format the data files, evaluate and predict. The file enzml_java_code.tar.gz contains the Java code used to format database data to ARFF and XML formats, to execute cross and train-test (jackknife) evaluations and to record evaluation results to database. More information is included in the readme.txt file and the Javadoc files. The code can be used with a MySQL database. To use a different database software, other JDBC drivers might be required. [file 1471-2105-13-61-S5.gz › java_code/utils/doc/uk/ac/ed/inf/utils/StringUtils.html]

StringUtils


---


|  |  |  |
| --- | --- | --- |
| **Overview** \| **Package** \| **Class** \| **Use** \| **Tree** \| **Deprecated** \| **Index** \| **Help** | |  |
| **PREV CLASS**   **NEXT CLASS** | **FRAMES**    **NO FRAMES**     **All Classes** |  |
| SUMMARY: NESTED \| FIELD \| CONSTR \| METHOD | DETAIL: FIELD \| CONSTR \| METHOD |  |


---


## uk.ac.ed.inf.utils Class StringUtils

```
java.lang.Object
  uk.ac.ed.inf.utils.StringUtils
```

---

``` public class StringUtils extends java.lang.Object ```

Class

**Version:**
:   11 Mar 2008

**Author:**
:   Luna De Ferrari luna.deferrari-at-ed.ac.uk

---

**Constructor Summary**

`StringUtils()`


| **Method Summary** | |
| --- | --- |
| `static java.lang.String` | `booleanTo01String(java.lang.Boolean booleanValue)`             Returns "1" if Boolean.TRUE, "0" if Boolean.FALSE |
| `static boolean` | `containsPositiveInteger(java.lang.String string)`             Returns true if the string contains a parsable integer |
| `static java.lang.String` | `fromInputStreamToString(java.io.InputStream in)` |
| `static boolean` | `isDash(java.lang.String string)`             check if the string is a dash |
| `static boolean` | `stringIsNullOrEmpty(java.lang.String string)` |

**Methods inherited from class java.lang.Object**

`equals, getClass, hashCode, notify, notifyAll, toString, wait, wait, wait`

**Constructor Detail**

### StringUtils

```
public StringUtils()
```


**Method Detail**

### booleanTo01String

```
public static java.lang.String booleanTo01String(java.lang.Boolean booleanValue)
```

:   Returns "1" if Boolean.TRUE, "0" if Boolean.FALSE

    :   **Parameters:**: `booleanValue` - **Returns:**: "1" if true, "0" if false

---


### containsPositiveInteger

```
public static boolean containsPositiveInteger(java.lang.String string)
```

:   Returns true if the string contains a parsable integer

---


### fromInputStreamToString

```
public static java.lang.String fromInputStreamToString(java.io.InputStream in)
                                                throws java.io.IOException
```

:   **Throws:**: `java.io.IOException`

---


### isDash

```
public static boolean isDash(java.lang.String string)
```

:   check if the string is a dash

---


### stringIsNullOrEmpty

```
public static boolean stringIsNullOrEmpty(java.lang.String string)
```

:   **Parameters:**: `string` - **Returns:**: true if a string is null or empty


---


|  |  |  |
| --- | --- | --- |
| **Overview** \| **Package** \| **Class** \| **Use** \| **Tree** \| **Deprecated** \| **Index** \| **Help** | |  |
| **PREV CLASS**   **NEXT CLASS** | **FRAMES**    **NO FRAMES**     **All Classes** |  |
| SUMMARY: NESTED \| FIELD \| CONSTR \| METHOD | DETAIL: FIELD \| CONSTR \| METHOD |  |


---
